# Footprint metabolomic profile shift of Sf9 insect cell line throughout growth and rabies VLP production

**DOI:** 10.1007/s00449-026-03428-1

**Published:** 2026-09-22

**Authors:** Júlia Públio Rabello, Júlia Dezanetti da Silva, Milena Miyu Teruya, Fernanda Angela Correia Barrance, Kevy Pontes Eliodório, Bárbara Castro, Thaissa Consoni Bernardino, Eutimio Gustavo Fernández Núñez

**Affiliations:** 1 Laboratório de Engenharia de Bioprocessos. Escola de Artes, Ciências e Humanidades (EACH), Universidade de São Paulo, Rua Arlindo Béttio 1000, São Paulo, SP CEP 03828-000 Brazil; 2 Laboratório de Desenvolvimento de Vacinas, Instituto Butantan, Av. Vital Brasil 1500, São Paulo, SP CEP 05503-900 Brazil; 3 IonMedicine, R. Apiacás, 247 - Sobreloja - Perdizes, São Paulo, SP CEP 05017-020 Brazil; 4 Laboratório de Biotecnologia Viral, Instituto Butantan, Av. Vital Brasil, 1500, São Paulo, SP CEP 05503-900 Brazil

**Keywords:** *Sf9* insect cell line, Footprint metabolomic, Target metabolomic, baculovirus, Virus-like particles, Rabies virus

## Abstract

The baculovirus/insect cell system is widely used for recombinant protein and virus-like particles (VLP) production, but its performance at high cell density is limited by infection-associated metabolic stress. In this study, a targeted metabolic footprinting approach was applied to characterize the extracellular metabolome of *Spodoptera frugiperda* Sf9 cells during baculovirus-mediated production of rabies VLP in chemically defined medium. Infected and uninfected cultures were cultivated in controlled bioreactor runs, and extracellular carbohydrates, amino acids, organic acids, and nucleosides were quantified by HPLC and LC-MS/MS. Metabolite data were converted into specific uptake and release rates, then also analyzed using Principal Component Analysis (PCA), Partial Least Squares Discriminant Analysis (PLS-DA), and Variable Importance in Projection scoring (VIP). Infection induced marked metabolic reprogramming, with increased glucose demand, dynamic shifts in amino acid consumption and release, and altered extracellular profiles over time. Although infected and control samples partially overlapped in PCA, supervised analysis improved group discrimination and identified sucrose, leucine, lysine, glycine, and pyruvate as the main variables associated with infection. Sequence-based compositional analysis of the recombinant proteins supported the relevance of amino acid-related metabolic changes, while transmission electron microscopy confirmed successful formation of rabies VLP with expected morphology. Together, these results provide a quantitative description of the metabolic burden imposed by rabies VLP production in Sf9 cells and highlight candidate metabolites for medium redesign and targeted feeding strategies to reduce the cell density effect and improve process productivity in baculovirus-based VLP manufacturing.

**Supplementary Information:**

The online version contains supplementary material available at https://doi.org/10.1007/s00449-026-03428-1.

## Introduction

The baculovirus/insect cell (B/IC) expression vector system has evolved from a niche tool to a mainstream instrument in the field of modern biotechnology [1]. It is renowned for its robust capacity to produce complex eukaryotic proteins, gene delivery vectors, and Virus-Like Particles (VLP) [2–4]. This system exploits the efficient protein synthesis machinery of insect cells [5, 6], including the extensively utilized *Spodoptera frugiperda* (Sf9) line, which are competent in carrying out the required post-translational modifications for the biological activity of numerous recombinant proteins [7].

There is robust evidence to validate the clinical and commercial viability of the B/IC platform, as exemplified by its utilization in the production of regulatory-approved vaccines, including Cervarix^®^ for human papillomavirus [8] and Provenge^®^ for prostate cancer [9], underscoring its significant impact on human health. Moreover, there are examples of approved vaccines for veterinary use [10]. A key application of this system is the generation of VLP, which are non-infectious, self-assembling structures that mimic the native conformation and high epitope density of viruses [11, 12]. This unique property makes them exceptionally potent at eliciting robust and specific immune responses [13], as evidenced by successful vaccines against influenza and SARS-CoV-2 [14].

Despite this proven potential, a persistent challenge in industrial bioprocessing remains the efficient scaling of production to meet demand, as the B/IC system faces difficulties in scalability and cost [15, 16]. Maximizing volumetric yield is best achieved by infecting cultures at high cell density. However, this approach is accompanied by a significant “cell density effect” [17], which is a major limiting factor, leading to a substantial decline in cell-specific productivity. This phenomenon indicates that the metabolic burden imposed by the baculovirus infection exceeds the cell’s biosynthetic capacity, likely due to nutrient depletion [18, 19]. Nevertheless, there are successful studies in increasing the cell density by using rational medium design and feeding strategy [20].

Acting as a potent metabolic engineer, the wild and genetically modified baculoviruses manipulate the host cell’s resources to fuel their own replication and the intense production of recombinant proteins [21, 22]. This redirection of cellular machinery leads to a substantial increase in the demand for energy [23], profoundly altering the host’s metabolic landscape [24]. Studies have shown that the metabolism of Sf9 cells post-infection is characterized by a heightened consumption of glucose, glutamine, and other amino acids, coupled with an increased production of metabolites like alanine [25].

Given the suitability of insect cells for VLP production and the efficiency of the B/IC system for protein expression, mitigating these metabolic limitations is essential. However, understanding the physiological state of cells in culture during infection and recombinant expression remains a complex challenge [26]. To this end, the analysis of extracellular metabolites through metabolic footprinting offers a powerful solution [27], providing a more robust and technically accessible means to monitor nutrient consumption and by-product secretion. The approach can be targeted, with prior selection of metabolites, or non-targeted, which comprehensively evaluates all metabolites without pre-selection [28]. However, non-targeted analysis generates a large volume of data that is complex to interpret and not always directly applicable to bioprocess engineering [29].

Cells secrete various metabolites into the culture medium, especially under unbalanced growth conditions, such as those induced by infection [30]. These secretions reflect cellular metabolic activity, allowing the physiological state to be inferred non-invasively. Furthermore, unlike intracellular metabolites requiring abrupt metabolic arrest for analysis, extracellular metabolites have a slower turnover, facilitating their detection [27]. For such an analysis, it is important to emphasize the significance of utilizing a Chemically Defined Medium (CDM). Unlike complex media derived from natural sources, CDM has a precisely defined chemical composition [31], providing a solid foundation for investigating extracellular components [32].

While the metabolic impact of baculovirus infection is recognized, a comprehensive, targeted analysis of the extracellular metabolome remains lacking. Most studies have focused on a limited set of nutrients (e.g., glucose, glutamine) [33, 34], leaving a significant gap in our understanding of the global metabolic rewiring [35]. Although global metabolic rewiring is a recognized feature of viral infection and replication, its specific metabolic consequences in the context of baculovirus-based VLP production remain insufficiently characterized. In this context, a targeted footprinting approach, capable of simultaneously quantifying a broad spectrum of metabolites, including carbohydrates, amino acids, organic acids, and nucleotides, might provide a more comprehensive view of how infection alters the extracellular metabolic environment. This methodology can identify not only which nutrients become limiting but also whether specific by-products accumulate [27], thereby providing additional information on the metabolic demands and adaptations associated with infection. It can contribute to a better understanding of the broader metabolic rewiring process, while complementary approaches (e.g., transcriptomics, intracellular metabolomics, flux analysis) would be required to elucidate its underlying molecular mechanisms.

Therefore, this work employs a targeted metabolic footprinting strategy to systematically compare the exo-metabolome of uninfected and baculovirus-infected Sf9 cells cultivated in a CDM. The concentrations were quantitatively profiled and calculated the consumption/production rates of a comprehensive set of metabolites: carbohydrates (sucrose and glucose) and amino acids/nucleoside/primary metabolites (Cystine, Aspartate, Asparagine, Serine, Alanine, Glutamate, Glycine, Glutamine, Threonine, Proline, Lysine, Histidine, Arginine, Gamma-aminobutyric acid **(**GABA), Valine, Methionine, Isoleucine, Leucine, Phenylalanine, Tryptophan, Taurine, Asymmetric dimethylarginine (ADMA), Guanosine, Tyrosine, Adenosine, Malate, Pyruvate, Lactate, Uric Acid, Succinate) in uninfected and infected cells.

Thus, the main goal of this work was to identify and quantify the specific alterations in the secretion/consumption metabolic profile induced by baculovirus infection of the *Sf9* cell line to produce Rabies-VLP (as an expression model), with the final aim of generating a quantitative dataset to guide the rational redesign of culture media. By characterizing temporal changes in nutrient concentration and potential inhibitory by-products, this research provides a foundation for developing optimized feeding strategies to overcome the cell-density effect and enhance the productivity of the baculovirus/insect cell system for the efficient production of VLP.

## Materials and methods

### Generation of recombinant baculoviruses

The constructions and transformations in bacteria were previously performed by the Viral Biotechnology Laboratory of the Butantan Institute. For the generation of recombinant baculoviruses that express the matrix protein and glycoprotein of the rabies virus, the pFastBacTM plasmid vector (Invitrogen, California, USA) was used, with translation controlled by the polyhedrin promoter, transformed into *Escherichia coli* DH10BacTM with the Bac-to-Bac system (Invitrogen, California, USA), following the manufacturer’s instructions and previously established protocol [36]. For the extraction and purification of the recombinant bacmid, the Maxiprep kit (Qiagen, Maryland, USA) was used [36, 37].

For insect cell transfection, 10 µg of the bacmid obtained was complexed with Cellfectin II^®^ cationic liposome (Invitrogen, CA, United States) in a 25 cm² T-flask with$\:\:5.0\:x\:1{0}^{5}$ Sf9 cells and the medium SF900™ III (Catalog number: 12658027, Thermo Fisher Scientific, NY, US) for 96 h at 28 °C. Then, the supernatant was clarified by centrifugation (5 min at 500 g) to obtain the recombinant baculoviruses BVG (Baculovirus carrying the Glycoprotein gene) and BVM (Baculovirus carrying the Matrix protein gene**)**. To propagate the infectious baculoviruses, another infection (second viral batch - L2) was accomplished in 100 mL Schott flasks with a working volume of 20 mL, at viable cell density of $\:1.0\:x\:1{0}^{6}$ Sf9 cells/mL and multiplicity of infection (MOI) of 0.1 plaque-forming units per cell (PFU/cell) in SF900™ III (Catalog number: 12658027, Thermo Fisher Scientific, NY, US). It was incubated for 96 h, followed by clarification and collection of the supernatant [38]. To propagate the number of infectious baculoviruses, the third viral batch (L3) was generated under the same conditions as L2 in a chemically defined medium, EX-CELL® CD Insect Cell Medium (Catalog number: 24381 C, Merck, Darmstadt, Germany).

### Cells and culture medium

For bioreactor inoculum, and Rabies-VLP generation, Sf9 insect cells, cultured in suspension in EX-CELL^®^ CD Insect Cell Medium were used. They were maintained at 28 °C under agitation at 110 rpm during viral propagation and inoculum preparation. Cultivation was initiated at a cell density of $\:1.0\:x\:1{0}^{6}\:$cells/mL, in a working volume of 20 mL, using 100 mL Schott flasks.

Viral quantification was then performed with Sf9 Easy Titer (Sf9-ET) cells, which were kindly provided by Ralph Hopkins and Dominic Esposito of the National Cancer Institute at Frederick (Maryland, USA). These cells were cultured in Sf900™ III medium supplemented with 2.5% fetal bovine serum (HyClone^®^, Cytiva, USA), also at an initial density of $\:1.0\:x\:1{0}^{6}\:$cells/mL in 20 mL culture medium, in 100 mL Schott flasks.

### Bioreactor runs

Two separate experiments were conducted under identical operating conditions, following a simple batch design in Minifors 2 bioreactors (1.5 L flask, Infors HT, Switzerland) with a nominal working volume of 500 mL. The culture conditions were maintained at 28 °C, with agitation at 80 rpm, dissolved oxygen (DO) concentration controlled at 30% air-saturation by automatic regulation of the gas mixture composition, and pH was not controlled. The inoculum for both experiments was standardized at a concentration of $\:5.0\:x\:1{0}^{5}$ cells/mL.

Viral infection is the only parameter differentiated between the two experiments. In one of them, the culture was infected at the time of infection (TOI) of 72 h (Cell Density at 4 * x* 10⁶ cel/mL) with viral batch L3, titrated at $\:1.0\:x1{0}^{8}$ PFU/mL and diluted in EX-CELL® CD culture medium (MOI of 3 for BVG and 2 for BVM). The second experiment, used as a control, remains uninfected. From each bioreactor run, 7 samples (5 mL volume) were collected at corresponding time intervals, yielding a total of 14 samples (*n* = 14), 7 for the Control group and 7 for the Infected condition (Table 1). In the case of the infection experiment, two samples were taken before and five after infection.

**Table 1:** Collected sample identification throughout both bioreactor runs, without (Cell growth) and with infection (VLP production). *

Sample identification	Assay	Sampling time from cell inoculation (h)	Time post-infection (h)		Phase
C1	Cell growth	0.0		–	Acceleration
C2		28.4		–	Acceleration/Exponential
C3		68.3		–	Exponential
C4		92.3		–	Exponential/Deceleration
C5		116.3		–	Deceleration
C6		139.9		–	Deceleration
C7		163.9		–	Deceleration
I1	VLP production	0.0		0	Acceleration
I2		68.6		0	Acceleration
I3		77.2		5.2	Infection
I4		92.7		20.7	Infection
I5		100.6		28.6	Infection
I6		116.9		44.9	Infection
I7		150.2		78.2	Infection

*The differences in sampling times were designed to ensure better characterization of each run based on its specific objective (growth or VLP production). For the VLP production run, greater emphasis was placed on the infection phase (5 samples); the other two samples—taken from the growth phase—were collected at the beginning and end of that phase, closely aligning with the corresponding samples in the growth run. The VLP production run was terminated early due to a drop in viability below 60%, which was established as the stopping criterion for this type of expression system [7]

### Cell growth data and viral titer

To quantify the concentration of viable cells (Xv) and cell viability (Cv), samples were diluted 10 times in phosphate-buffered saline (PBS) and stained with trypan blue 0.04%. Cell counting was performed by optical microscopy using a Neubauer chamber, following the dye exclusion method [39]. The corresponding cell growth and viability curves are presented in the Supplementary Material as Figs. S2 and S3, respectively.

The titer of infectious baculovirus was determined by a 96-well plate titration assay [40]. For this assay, the Sf9 ET cell line was used, which were genetically modified to express green fluorescent protein (eGFP) under the control of the baculovirus polyhedrin gene promoter. When infected, these cells emit fluorescence, allowing the quantification of infectious viral particles. Based on the absence or presence of fluorescence, the titers of infectious baculovirus expressed in TCID_50_ mL^− 1^ were calculated according to a well-established protocol [41]. It was also possible to determine the number of plaque-forming units per mL (PFU/mL) considering the PFU/TCID_50_ mL^− 1^ ratio equal to 0.69 [42].

### Transmission electron microscopy

The morphological characterization of the VLP was performed by transmission electron microscopy (TEM), following a previously established protocol based on the specific immunolabeling of the rabies virus G and M proteins [38]. Briefly, clarified supernatants (45 mL) were ultracentrifuged (80,000 * x* g, 3 h), and pellets were resuspended overnight in 500 µL PBS. Samples on carbon-coated 300-mesh copper grids were immunogold-labeled with anti-RVGP (LifeSpan BioSciences, USA) and anti-RVM (Cusabio, China) diluted 100-fold, followed by gold-conjugated secondary antibodies: 10 nm anti-mouse (A31561, 1:50, Thermo Fisher, USA) and 5 nm anti-rabbit (A31565, Thermo Fisher, USA), 1:50). After three PBS washes, grids were stained with 2% uranyl acetate for 1–2 min and analyzed by transmission electron microscopy (JEM-1011, JEOL, Japan).

Particle size was determined from TEM micrographs through manual evaluation performed by trained analysts. Particles were selected according to predefined morphological characteristics to differentiate intact VLP from nonspecific cellular material and other background structures. A total of 20 VLP that were clearly separated from neighboring structures and displayed both immunogold labels were selected for dimensional analysis. The 10 nm and 5 nm gold nanoparticles corresponded to G and M protein, respectively. Measurements from these 20 dual-labeled particles (*n* = 20) were subsequently used to characterize the representative mean diameter and size distribution of the assembled VLP within the unpurified sample matrix.

### Enzyme-linked immunosorbent assay (ELISA) for protein g and western blot semi quantification for protein M

Protein G was quantified by ELISA using a Rabies Glycoprotein Enzyme Immunoassay kit (Pasteur Institute, Paris, France), as previously described in [43]. A 96-well plate was coated with 200 µL of Mab-D1 primary antibody diluted 1:2000 in carbonate buffer (50 mM NaHCO₃ buffered with 50 mM Na₂CO₃, pH 9.6) and incubated for 16 h at 4 °C. The plate was then blocked with 300 µL of blocking solution (5% sucrose w/v and 3% BSA w/v in carbonate buffer) for 30 min at 37 °C. The plate was washed five times with 1X PBS containing 0.05% Tween 20 (washing buffer), followed by the addition of 150 µL of either the standard curve (inactivated and lyophilized rabies virus provided in the kit) or sample supernatant, in duplicate. The plate was incubated for 1 h at 37 °C and subsequently washed six times with washing buffer. Then, 200 µL of peroxidase-conjugated primary antibody MAb-D1-Po (Pasteur Institute, Paris, France), diluted 1:2000 in conjugate buffer (1% BSA w/v and 0.05% Tween 20 v/v in 1X PBS, pH 7.0), was added and incubated for 1 h at 37 °C. After six additional washes with washing buffer, 200 µL of chromogenic substrate (2 mg/mL orthophenylenediamine and 1 µL/mL H₂O₂ in citrate buffer containing 45 mM sodium citrate and 16.3 mM anhydrous citric acid, pH 5.6) was added. The plate was incubated for 30 min at room temperature and protected from light. The reaction was stopped by adding 50 µL of 4 N sulfuric acid. Absorbance was measured at 492 nm using a Multiskan EX microplate reader (Thermo Scientific, Waltham, MA, USA).

Western blot analysis was performed to detect and semi-quantify Protein M in culture supernatants and a positive control sample (inactivated rabies virus). For sample preparation, 15 µL of culture supernatant was mixed with 5 µL of loading buffer. Inactivated rabies virus (125 ng/mL) was included as a positive control, while 3 µL of Spectra™ Multicolor Broad Range Protein Ladder (Thermo Fisher, USA) was used as a molecular weight reference. Protein separation was performed by electrophoresis at a constant voltage of 200 V until the molecular weight marker bands were adequately resolved. Proteins were then transferred to a 0.45 μm nitrocellulose membrane (GE Healthcare, USA) at 10 V for 2.5 h. The membrane was blocked with Pierce™ Clear Milk Blocking Buffer (37587, Thermo Fisher, USA) for 1 h to prevent nonspecific binding. After blocking, the membrane was washed three times with PBS containing 0.05% Tween^®^ 20, with each wash performed for 10 min. Protein M was detected using a rabbit anti-Protein M primary antibody (1:4000, PA5-117558, Thermo Fisher Scientific, USA). Following three additional 10-min washes with PBS containing 0.05% Tween^®^ 20, the membrane was incubated for 1 h with an HRP-conjugated anti-rabbit secondary antibody (1:5000, 31460, Thermo Fisher, USA). Then, the membrane was washed three times with PBS for 10 min each. Detection was performed using the SuperSignal™ West Pico PLUS Chemiluminescent Substrate Kit (34580, Thermo Fisher Scientific, USA), and the resulting signal was recorded using an ImageQuant™ LAS 500 system (GE Healthcare, USA).

### Metabolomic profile data

#### Sample preparation

Following thawing, a 100 µL aliquot of each sample was transferred to Falcon-type tubes and diluted with 9.9 mL of LC-MS grade water, yielding a 100-fold dilution of the original fraction. From this solution, 1 mL was transferred to Eppendorf tubes and mixed with 10 µL of a derivatizing solution consisting of N-ethylmaleimide (200 µM) and citric acid (2 mM), both prepared in LC-MS grade methanol. The mixture was homogenized and incubated for 1 h at room temperature. The derivatized samples were filtered through 0.22 μm PTFE syringe membranes. Subsequently, 50 µL of the filtrate was transferred to a 96-well plate, to which 200 µL of a protein precipitation solution composed of acetonitrile, isopropanol, and formic acid (69:30:1, v/v/v) was added. This solution contained an isotopically labelled internal standard mix comprising amino acids, carnitines, organic acids, and nucleosides. The plate was vortexed for 10 min, cooled to − 20 °C for 10 min, and centrifuged at 3000 rpm for 10 min at 10 °C. The supernatant was transferred to a new shallow-well plate and diluted 1:1 with ultrapure isopropanol/water (3:7, v/v) prior to instrumental analysis.

#### Determination of non-sugar analytes by LC-MS/MS

Extracellular analytes (Cysteine, Aspartate, Asparagine, Serine, Alanine, Glutamate, Glycine, Glutamine, Threonine, Proline, Lysine, Histidine, Arginine, GABA, Valine, Methionine, Guanosine, Tyrosine, Adenosine, ADMA, Isoleucine, Leucine, Phenylalanine, Tryptophan, Taurine, Pyruvate, Malate, Lactate, Uric acid and Succinate) analysis was performed using liquid chromatography coupled with tandem mass spectrometry (LC-MS/MS). Chromatographic separation was achieved on a Discovery HS F5 column using a binary mobile phase system consisting of water with 0.1% formic acid (Mobile Phase A) and acetonitrile with 0.1% formic acid (Mobile Phase B). A 5 µL injection volume was used for all samples. Detection was carried out in multiple reaction monitoring (MRM) mode, with transitions optimized for the specific analysis of primary metabolites. Quantification was based on analytical calibration curves constructed from reference standards at known concentrations, and analyte identification was confirmed by comparing the specific MRM transitions and retention times of the samples with those of the authentic standards.

#### Determination of glucose and sucrose

Glucose and sucrose concentrations in infected and uninfected samples were quantified by a high-performance liquid chromatography (HPLC) system (Shimadzu Prominence LC-20AB, Japan) equipped with a refractive index detector, in accordance with the established protocol [44]. Before injection, all samples were clarified by centrifugation to remove cells and cell debris. The resulting supernatants were then diluted tenfold with ultrapure water and filtered through 0.22 μm syringe filters. Chromatographic separation was achieved using a Bio-Rad Aminex HPX-87 C column maintained at 85 °C, with ultrapure water as the mobile phase at a flow rate of 0.6 mL·min⁻¹. Quantification was performed using external standard calibration, with linear calibration curves prepared for each analytical batch.

#### Determination of uptake and release specific rates

Uptake and release specific rates were calculated using two distinct approaches depending on the experimental condition and sampling time. For the samples of uninfected cells collected during the acceleration, exponential and deceleration phases, specific rates were calculated according to Eq. 1, where C represents the analyte concentration (mmol/L), X_v_ is the viable cell density (cells/L), $\:{\upmu\:}$ is the specific growth rate (h^− 1^) and $\:{\mathrm{Y}}_{\frac{\mathrm{A}}{{\mathrm{X}}_{\mathrm{V}}}}$ is the analyte-viable cell conversion yield (nmol 10^− 6^ cell^− 1^). According to the sign convention adopted, positive values obtained from Eq. 1 were interpreted as net production of the component, whereas negative values were interpreted as net consumption. Metabolite evaporation or degradation was not considered.

1 $$\:{\mathrm{q}}_{C}=\frac{\mathrm{d}\mathrm{C}}{\mathrm{d}\mathrm{t}}\:\bullet\:\:\frac{1}{{\mathrm{X}}_{\mathrm{V}}}={\upmu\:}\bullet\:{\mathrm{Y}}_{\frac{\mathrm{A}}{{\mathrm{X}}_{\mathrm{V}}}}$$

For all samples collected in the experiment with infection over post-infection phase, specific rates ($\:{\mathrm{q}}_{{\mathrm{c}}_{\mathrm{n}}}$) were calculated using a midpoint approximation between two consecutive sampling points, as described in Eq. 2. The same sign convention was applied to values obtained from Eq. 2.

2 $$\:{\mathrm{q}}_{{\mathrm{c}}_{\mathrm{n}}}\left(\stackrel{-}{\mathrm{t}}\right)=\frac{{\mathrm{C}}_{\mathrm{n}-}{\mathrm{C}}_{\mathrm{n}-1}}{{\mathrm{t}}_{\mathrm{n}-}{\mathrm{t}}_{\mathrm{n}-1}}\:\bullet\:\:\frac{1}{\stackrel{-}{\mathrm{X}\mathrm{v}}}$$

Where $\:\stackrel{-}{\mathrm{t}}$ is the average of the two consecutive sampling times ($\:{\mathrm{t}}_{\mathrm{n}\:,\:}{\mathrm{t}}_{\mathrm{n}-1}$); $\:{\mathrm{C}}_{\mathrm{n}}$ is the concentration of the analyte at $\:{\mathrm{t}}_{\mathrm{n}}$ and $\:{\mathrm{C}}_{\mathrm{n}-1}$ is equivalent for $\:{\mathrm{t}}_{\mathrm{n}-1}$, $\:\stackrel{-}{{\mathrm{X}}_{\mathrm{V}}}$ is the viable cell density average between $\:{\mathrm{t}}_{\mathrm{n}\:,\:}{\mathrm{t}}_{\mathrm{n}-1}$.

### Data processing and normalization

Following imputation, a three-step normalization procedure was applied to address the wide concentration range across metabolites and render the variables comparable for multivariate analysis. First, samples were normalized by sum because the samples share similar overall metabolite profiles, and this step preserves relative proportions while adjusting for differences among samples [45]. Next, a log base-2 transformation was applied to compress the concentration scale, approximate symmetrical distributions, and reduce the influence of extreme values, which is suitable for data tending toward a log-normal distribution [46]. Finally, range scaling was used, as it compares metabolites relative to the biological response range while equally weighing the importance of all metabolites, it is an approach recommended when metabolite ranges differ substantially [47]. The result of this normalization is available in Fig. S1 – Supplementary material.

#### Principal component analysis (PCA)

A regular (two-components) and 3-dimensional PCA were employed to visualize the similarity or divergence between samples and bioreactor runs (with and without infection) based on the normalized concentration of analytes. For this purpose, the software MetaboAnalyst 6.0 (Wishart Research Group, Alberta, Canada) was used, following the guidelines provided on the website (https://www.metaboanalyst.ca). The input data matrix consisted of normalized concentration values, with rows corresponding to individual samples and columns corresponding to the analyzed metabolites. The similarity between the metabolic profiles of the samples was represented by the spatial proximity of their points on the principal component scores plot.

#### VIP obtained via PLS-DA

To identify the metabolites that contributed the most to the discrimination between the experimental groups, PLS-DA was employed. This supervised technique maximizes the covariance between the metabolite abundance data (X-matrix) and the categorical variable defining the groups (Y-matrix), allowing for better separation between predefined classes and facilitating the identification of metabolically relevant features. The PLS-DA model and biplot were also constructed using the multivariate analysis module of the MetaboAnalyst 6.0 platform, utilizing the same input data as the PCA.

Model validation was performed by the software itself using the Leave-One-Out Cross-Validation (LOOCV) tool. Following model validation, the importance of each metabolite for group discrimination was quantified using the VIP score. The VIP score synthesizes a variable’s contribution to the PLS-DA model, considering both the weights and the explained variance in each component. As a criterion, metabolites with a VIP score ≥ 1.5 were considered the most influential for distinguishing between the groups and was therefore selected for subsequent investigation regarding their potential role.

### Sequence retrieval and compositional analysis

To further investigate the results of those amino acids results with VIP score ≥ 1.5, primary protein structures of the Rabies virus glycoprotein (G) and matrix protein (M) were obtained from the Universal Protein (UniProt) database using BLAST (Basic Local Alignment Search Tool). The reference sequences with the highest percent identity and query coverage were selected for analysis (Accession numbers Q85422 and P0DOF2). For each sequence, the linear order of amino acid residues was treated as a string of single-letter codes, which were counted in Excel to register the frequency of each of the 20 standard amino acids individually. Due to a 3:2 stoichiometric ratio between the glycoprotein and matrix protein, the raw amino acid counts were adjusted proportionally to reflect the actual amino acid required composition.

## Results and discussion

Before presenting the results, it is important to address a key characteristic of the infection and VLP production process that directly impacts metabolomic analysis in this work. The infection with baculovirus leads to cellular lysis [48], which, while necessary for efficient VLP recovery, has an important drawback: all intracellular contents are released into the culture medium alongside the VLP. Since this study compares the metabolic footprint profiles of Rabies virus-infected Sf9 cells producing VLP versus healthy controls, this lysis can introduce confounding signals. That means some analytes that should not increase under normal conditions (e.g., amino acids and sugars) may appear elevated at certain time points due to passive leakage from lysed cells, rather than active metabolic secretion.

For an honest comparison of concentration in the samples, the specific uptake and release rate will be used as criteria. Nonetheless, the brute values of concentration for every measured analyte for both conditions plotted during the full hours of the experiment are in Fig. S4–34 of the Supplementary Material. All monitored analytes, their Kyoto Encyclopedia of Genes and Genomes Identifiers (KEGG IDs), and abbreviations used in this paper are listed in Table 2.

**Table 2:** Monitored analytes’ names, KEGG IDs, and abbreviations

Analyte name	Abbreviation	KEGG ID	Analyte name	Abbreviation	KEGG ID
Cysteine	Cys	C00097	Guanosine	Guo	C00387
Aspartate	Ast	C00049	Tyrosine	Tyr	C00082
Asparagine	Asn	C00152	Adenosine	A	C00212
Serine	Ser	C00065	ADMA	–	C03626
Alanine	Ala	C00041	Isoleucine	Ile	C00407
Glutamate	Glu	C00025	Leucine	Leu	C00123
Glycine	Gly	C00037	Phenylalanine	Phe	C00079
Glutamine	Gln	C00064	Tryptophan	Trp	C00078
Threonine	Thr	C00188	Taurine	Tau	C00245
Proline	Pro	C00148	Pyruvate	Pyr	C00022
Lysine	Lys	C00047	Malate	Mal	C00149
Histidine	His	C00135	Lactate	Lac	C00186
Arginine	Arg	C00062	Uric acid	UA	C00366
GABA	–	C00334	Succinate	Scn	C00042
Valine	Val	C00183	Sucrose	Suc	C00089
Methionine	Met	C00073	Glucose	Glu	C00031

### Comparison of consumption and production for analytes

Consumption and production rates for every analyte for the control run and pre-infection stage in infection assay were calculated (Table 3). Those values reflect a common metabolic activity without perturbation. In contrast, Table 3 shows how the consumption and production profiles change following infection, highlighting potential shifts between the maximum and minimum calculated values; the calculation for every time interval is available in Supplementary Material Table S1.

**Table 3:** Specific consumption and production rates ($\:\mathrm{x}$ nmol 10^− 6^ cell^− 1^ h^− 1^) for uninfected cells assay over different growth phases and cells before infection (pre-infection)

Analyte	Acceleration	Exponential*	Pre-infection*	Deceleration	Analyte	Acceleration	Exponential*	Pre-infection*	Deceleration
Cys	− 2.84	− 0.19	− 0.30	− 0.24	Guo	0.11	0.01	0.01	− 0.002
Ast	− 40.75	− 5.81	0.82	− 1.75	Tyr	− 12.90	− 0.67	− 1.29	− 0.93
Asn	− 8.25	− 0.60	1.98	− 1.61	A	− 0.02	0.0028	− 0.0004	− 0.001
Ser	− 36.11	− 8.08	− 9.47	− 4.97	ADMA	0.01	0.01	0.01	0.01
Ala	125.36	38.43	37.34	4.03	Ile	− 23.73	− 0.85	− 2.73	− 2.01
Glu	− 25.79	3.77	3.00	− 9.24	Leu	− 13.41	− 2.01	− 3.47	− 2.41
Gly	− 10.78	0.12	− 0.75	− 0.68	Phe	− 26.31	3.02	− 3.47	− 1.44
Gln	− 5.16	− 21.03	17.03	− 17.14	Trp	− 9.80	− 0.28	− 1.09	− 0.90
Thr	− 13.93	0.50	− 3.34	− 2.55	Tau	− 1.75	0.18	− 0.10	− 0.03
Pro	− 11.35	− 1.48	− 5.59	− 1.02	Pyr	5.16	0.08	− 7.73	0.59
Lys	− 58.81	− 0.76	− 0.82	− 0.86	Mal	− 0.52	− 0.47	− 0.10	− 0.23
His	− 9.60	0.87	0.07	− 0.72	Lac	6.71	− 0.80	− 0.41	0.05
Arg	5.16	− 3.99	− 8.65	− 2.61	UA	− 85.63	− 0.60	3.27	2.65
GABA	− 0.06	0.01	0.01	0.002	Scn	− 4.90	− 0.15	− 0.19	0.04
Val	− 35.08	0.23	− 2.66	− 1.68	Suc	− 13.93	− 6.63	1.43	− 2.45
Met	− 24.76	0.51	− 3.54	− 2.26	Glc	− 51.54	− 48.46	− 19.10	− 31.96

*Comparable

Sample identification for each growth phase is provided in Table 1. The Pre-infection column correspond to I1 and I2

As shown in Table 3, there is a column from the infection experiment, called “Pre-infection” it presents data from the time interval before infection, that is, before the 72 h mark TOI. As a criterion for choosing the correct phase for comparison, the µ was calculated for each time interval of interest. For the control experiment, the µ values were: 0.00903 h⁻¹ during the acceleration phase, $\:{{\upmu\:}}_{\mathrm{m}\mathrm{a}\mathrm{x}}$=0.02547 h⁻¹ during the exponential phase, and 0.02079 h⁻¹ during the deceleration phase. For the infection experiment, the $\:{{\upmu\:}}_{\mathrm{m}\mathrm{a}\mathrm{x}}$ calculated for the first time interval (before infection) was 0.03028 h⁻¹. Comparing these values, the exponential phase of the control experiment was chosen as the comparable column, as it is closest in value to the infection experiment’s pre-infection. Moreover, *μ* values for both groups were consistent with the literature for Sf9 cells for all phases [17].

An overall pattern can be seen in Table 4, $\:{\mathrm{q}}_{C}$ in the acceleration phase is much higher than in other phases. During this phase, cells are highly metabolically active and are adjusting to the specific conditions of the growth media; they consume significant resources, even though the total cell number does not increase much [49]. Thus, as they are gathering and preparing resources to start the exponential phase, when cell concentration will increase, $\:{\mathrm{q}}_{C}$ should indeed be higher in those conditions, especially for amino acids/energy sources consumption and by-products production. Additionally, it is also clear that infection not only shifts averages but also increases variability and extreme values.

**Table 4:** Maximum and minimum calculated specific consumption and production rates ($\:\mathrm{x}$ nmol 10^− 6^ cell^− 1^ h^− 1^) for the infected cells assay after infection

Analyte	Maximum	Minimum	Analyte	Maximum	Minimum
Cys	0.15	− 0.74	Guo	0.16	− 0.01
Ast	9.74	− 6.73	Tyr	1.19	− 2.24
Asn	16.06	− 23.09	A	0.009	− 0.004
Ser	13.15	− 14.71	ADMA	− 0.02	0.03
Ala	44.48	14.96	Ile	8.98	− 5.00
Glu	6.94	− 24.69	Leu	2.85	− 7.50
Gly	3.24	− 2.24	Phe	5.77	− 4.64
Gln	19.41	− 147.52	Trp	4.81	− 3.83
Thr	1.91	− 9.30	Tau	0.26	− 0.40
Pro	9.30	− 3.29	Pyr	5.50	− 9.05
Lys	2.00	− 6.77	Mal	0.56	− 2.37
His	1.66	− 4.23	Lac	6.28	− 1.32
Arg	1.90	− 5.77	UA	28.27	− 36.11
GABA	0.02	− 0.01	Scn	1.09	− 1.26
Val	3.53	− 2.94	Suc	− 0.57	− 15.07
Met	5.23	− 6.58	Glc	− 4.22	− 103.73

*Sf9* cells mainly utilized glucose as a carbon source, although they also metabolized other more complex sugar sources, sucrose, but at a much lower rate than in previous work [50]. This is expected because insect cells need to break down Sucrose into glucose and fructose before producing energy and any needed molecules for the cells’ maintenance and growth [51]. Therefore, utilizing the ready-to-use supply is preferable.

Still, a higher consumption of energy sources was already expected from the infected group because the virus is increasing the demand for pools of free nucleotides, needed to replicate the virus, and for amino acids, required for the production of recombinant proteins [52] as well as energy for simple viability maintenance. Part of this prediction was observed in this work, nucleotides such as Adenosine and Guanosine had no relevant fluctuations in $\:{\mathrm{q}}_{C}$, but the consumption rate for glucose for the infected group had a significant increase after the TOI, as in [53], while sucrose consumption rate decreased. Also, since glucose’s $\:{\mathrm{q}}_{C}$ is significantly higher in the infected group than in the control, it can be concluded that energy demand did indeed increase. However, despite this higher demand, the preference for monosaccharides remained unchanged. It is important to note that even in a significantly higher glucose $\:{\mathrm{q}}_{C}$, the carbon and energy source was not limited. The final concentration of glucose remained higher than 30 mmol/L (Supplementary material, Fig. S12).

Other works also imply that Sucrose consumption is small or non-existent in *Sf9* cells, especially in the presence of glucose [54], justifying why similar research do not even measure its consumption by this cell line [53]. However, in this similar work [50], sucrose consumption is said to be a good marker between infected and control groups since it increased almost 3-fold after infection, this issue will be addressed later in this work.

Glucose can serve as an energy source in two main ways: its complete oxidation or it can be converted to alanine or glutamate, via pyruvate and α-ketoglutarate [55]. Consequently, as the infected group had higher consumption rate of glucose, it is feasible to expect that it produced more alanine, glutamate and GABA, since the last is produced using glutamate. Although very subtle, this prediction also appears in this work. Alanine and GABA were indeed produced in larger quantities by infected cells. In fact, alanine is the main byproduct of insect cell metabolism; these cells release more nitrogen in the form of alanine than in the form of ammonia or uric Acid [54], but no inhibitory effect related to alanine was reported [19]. Alanine’s production is highly dependent on glucose metabolism, proximately 67% [56], which means that the full energetic potential of the sugar was not used since it was not oxidized. So, to produce alanine, insect cells waste energy. Such waste has been reduced by rational feeding strategies [55].

The undulatory characteristics of glutamate consumption/production make this analysis more complex. This amino acid production is the result of glutamine’s metabolism, which is further fed into the tricarboxylic acid (TCA) cycle [53]. Glutamine is described as a main nutrient for insect cells, serving as a key metabolite supporting rapid growth and energy production, particularly in cell culture systems [57]. Besides its consumption, this amino acid can also be synthesized if there is available ammonia [56], which could explain why, sometimes, its quantity increases and decreases during infection. Overall, glutamine was more consumed than produced in both groups, it aligns with previously cited work’s findings.

Lactate exhibited a more complex and dynamic behavior, alternating between production and consumption. This pattern is consistent with previously described metabolic behavior in cultured cells, where lactate is initially produced during active growth as a byproduct of glycolysis, contributing to medium acidification but also not inhibitory [58]. As reported in Sf9 cells, Lactate accumulation occurs while glucose is available, but upon glucose depletion, lactate is subsequently consumed as an alternative carbon source [54]. In this context, the variability in lactate observed in the infected condition likely reflects an unstable balance between glycolytic overflow and lactate reutilization. This is further supported by the presence of pyruvate fluctuations and constant alanine production, indicating that pyruvate could be preferentially redirected toward alanine via transamination rather than accumulating or being fully reduced to lactate.

Beyond the initial description of pyruvate redirection toward acetyl-CoA [59], a subsequent study [60] quantitatively resolved the fluxes through both the standard and alternative pathways. This alternative route is particularly relevant because Sf9 cells require pyruvate and TCA intermediates to sustain mitochondrial redox potential and adequately support O₂ delivery for aerobic metabolism. This work also suggests that the formation of pyruvate is a consequence of the high rate of incorporation of amino acids into the TCA cycle. However, as many important molecules in those processes were not measured in this work, besides succinate and malate, it is highly difficult to understand fully the extent of the $\:{\mathrm{q}}_{C}$ values and fluctuations for pyruvate.

Other works [60] also monitored relevant amino acids in the TCA cycle for infected with baculovirus Sf9 cells and studied how the infection changed its metabolism. In this work, the authors were able to quantify the relative contribution of the different amino acids to oxidation in the TCA cycle. The most relevant contributions, in order of percentage, came from Gln/Glu, Val, Leu, Ast/Asn, Lys and Ile.

In this work, infected cultures $\:{\mathrm{q}}_{C}$ for amino acid showed significant temporal variability, with net synthesis interleaved with periods of high consumption. Aside from the potential of quantification of the amino acids present in the media following cell lysis, this dynamic behavior might indicate loss of steady-state regulation [61], in which amino acid utilization shifts over time in response to changing cellular demands during infection. In contrast to highly dynamic amino acids such as glutamine and asparagine, analytes including GABA, cysteine, and histidine exhibited relatively low flux variability across the infection time course, suggesting that some metabolic pathways may remain tightly regulated despite metabolic reprogramming.

### Overview of comparative analysis

The primary goal of statistical analysis is the categorization and prediction of sample properties through the generation of models that capture information contained in data by reducing data dimensionality into inferred variables, thereby helping to identify major trends and features [62]. However, the quality of statistical models depends significantly on the choice of data pre-processing, scaling, and normalization methods. Successful data analysis requires careful investigation of multiple models to build consensus; in this study, many combinations of data processing were tested before arriving at the final results.

#### PCA

**Fig. 1:**
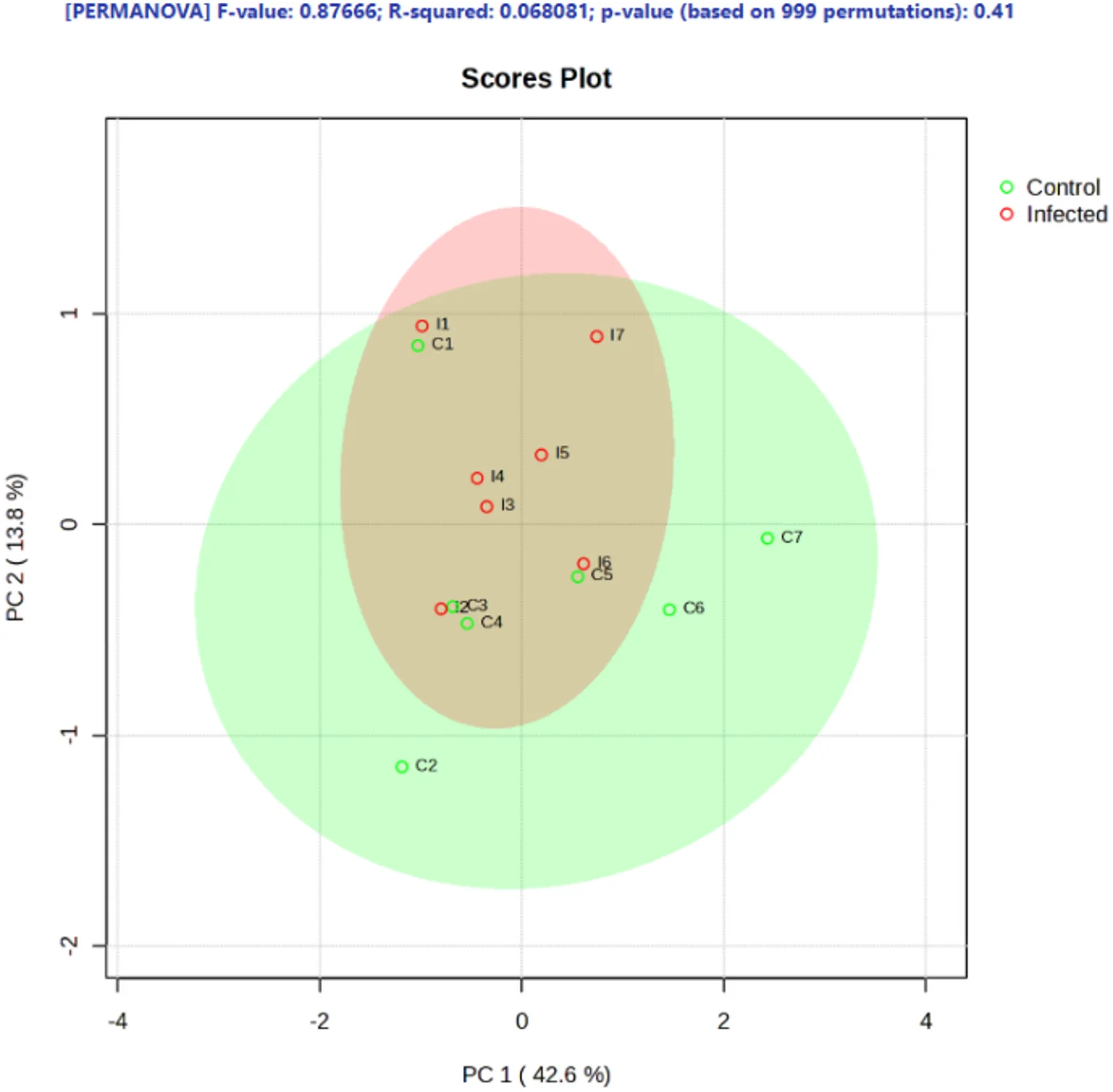
PCA score plot illustrating clustering patterns based on metabolic similarities among the samples, with each point representing an individual observation. Ellipses denote the 95% confidence intervals for each experimental group. C for Control and I for infected. For sample codification, see Table 1

PCA (Fig. 1) was conducted to investigate clustering patterns in the metabolic profiles of infected cells and the control group. The analysis revealed an almost total overlap between control and infected samples along PC1 (42.6%) and PC2 (13.8%). However, some sampling points were placed as far as possible from each other, especially at the final stages of the experiment. A partial overlap was expected because not every analyte is relevant to the infection process. It means that they are not related to the component being studied, which introduces irrelevant variation and compromises data analysis [63].

**Fig. 2:**
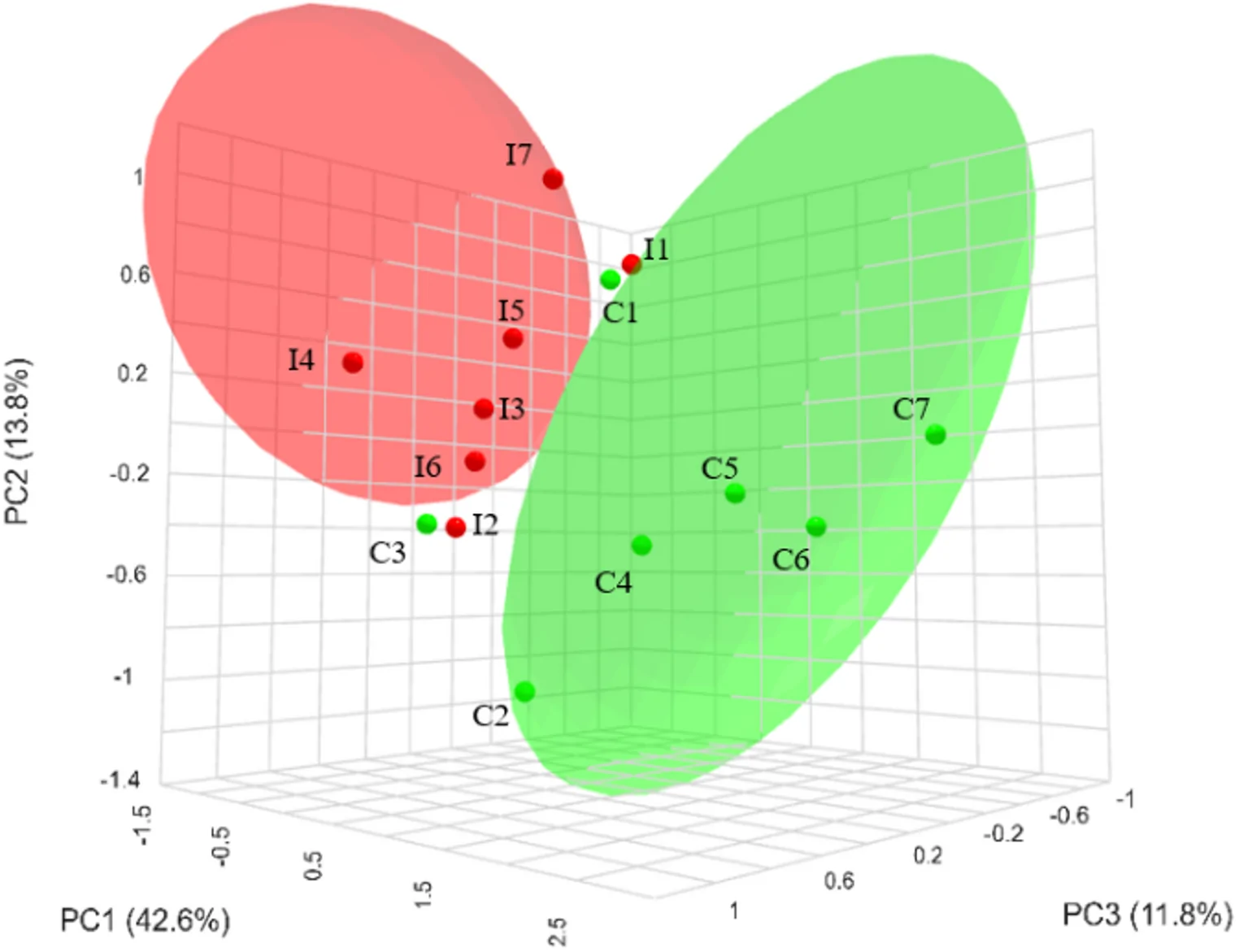
3D PCA score plot illustrating clustering patterns based on metabolic similarities among the samples, with each point representing an individual observation. Ellipses denote the 95% confidence intervals for each experimental group. C for Control and I for Infected. For sample codification, see Table 1

In the 2D plot, the Control and Infected groups appear almost totally overlapping, indicating that the first two principal components (56% of the total variance) are insufficient to capture the relevant biological difference between groups. A 3D analysis was necessary because the separation between groups could be contained in higher-order components (PC3 and beyond), which account for the remaining 44% of the data variance, the result is shown in Fig. 2.

As shown in the 3D PCA score plot (Fig. 2), only two observations lie outside the 95% confidence ellipsoid; both correspond to the time‑zero samples from each experimental replicate. The sole red point within the green ellipsoid represents the pre‑infection time point, aligning precisely with its expected position. The three‑dimensional representation offers noticeably better discrimination between groups than the two‑dimensional equivalent and captures almost 70% of data variation.

#### Partial least squares discriminant analysis (PLS-DA)

**Fig. 3:**
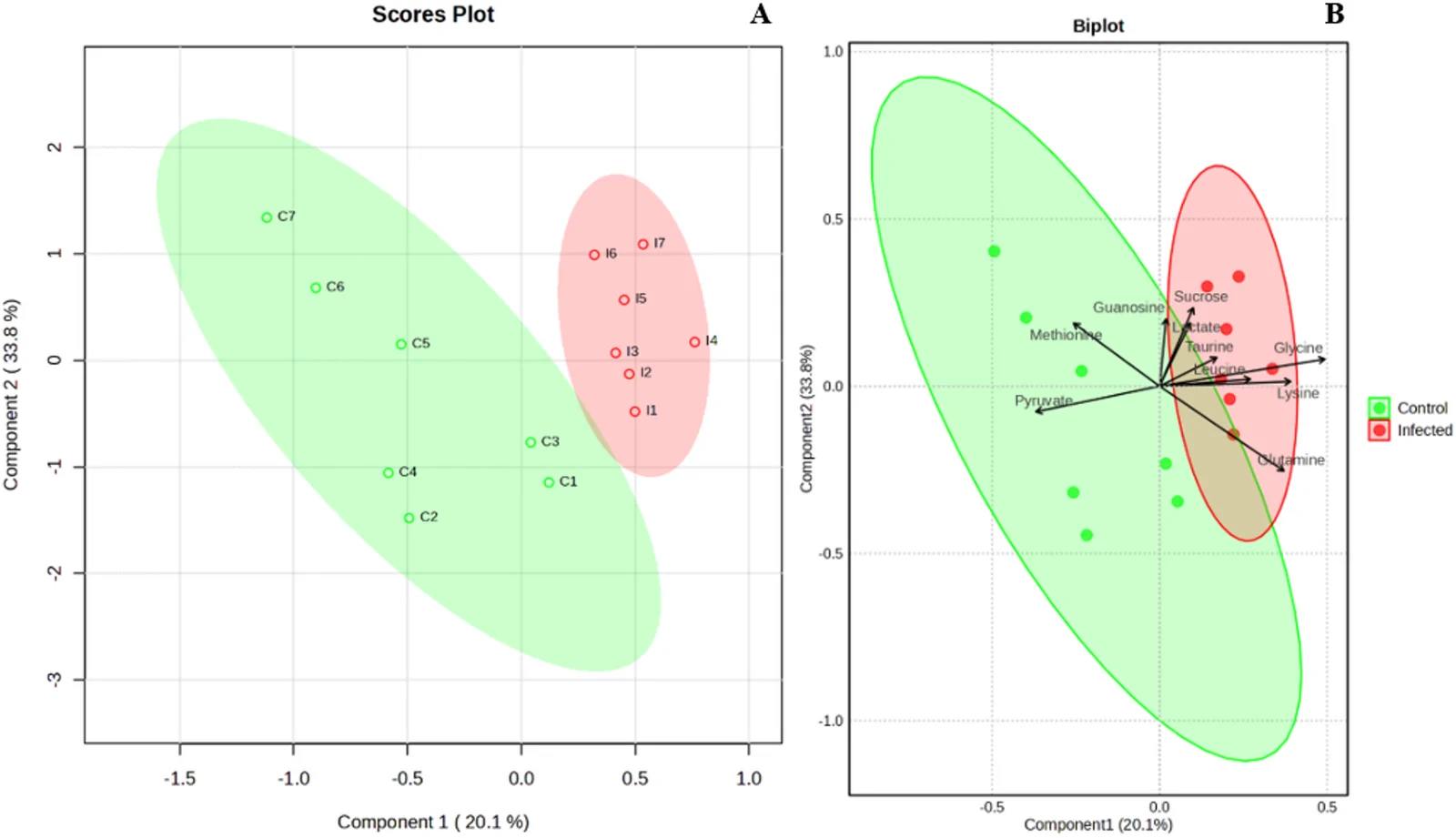
Partial least squares discriminant analysis (PLS-DA). **A**. Scores plot of metabolic profiles from control (green) vs. infected (red) groups with sample names. **B**. PLS-DA scores and loadings (metabolites) biplot. C for Control and I for infected. For sample codification, see Table 1

Even though MetaboAnalyst can generate a biplot based solely on PCA, the enhanced approach using a three-dimensional analysis revealed the need for a different strategy. PLS-DA was selected because it performs dimensionality reduction while fully incorporating class label information [64]. With the groups now almost completely separated, as shown in Fig. 3A, a biplot could be meaningfully applied, as presented in Fig. 3B.

The PLS-DA scores plot (Fig. 3A) demonstrates a clear separation between the Control and Infected groups, primarily along Component 1 (20.1%), with infected samples clustering toward positive values and control samples toward negative values. This separation is considerably more defined than that observed in the two-dimensional PCA, which can be explained by the distinct objectives of the two methods. PCA is an unsupervised approach that captures the major sources of overall variance in the dataset without considering the predefined experimental groups [62]. Therefore, the main principal components do not necessarily capture the variation associated with group discrimination, and separation between groups may only become apparent when additional components are considered. In contrast, PLS-DA is a supervised approach that incorporates class label information and identifies latent variables that maximize the covariance between metabolite profiles and the predefined experimental groups [64]. Thus, the clearer separation observed in PLS-DA is expected and reflects its ability to emphasize variation associated with class discrimination rather than a greater capacity to capture the overall variance of the dataset. The 95% confidence ellipses show minimal overlap, supporting the robustness of the metabolic differences between groups. Notably, the control group exhibited greater dispersion along both components, suggesting increased temporal metabolic variability. There is a clear trend in Fig. 3A, where samples are displayed vertically in approximate order of sampling time, from bottom to top, especially for the infected group. This shows that Component 2 (33.8%) can be related to a certain capture of an important part of the process: the effect of time.

The corresponding biplot (Fig. 3B) reveals the metabolites most contributing to group separation. sucrose, glycine, lysine, taurine, glutamine, lactate, and guanosine present loading vectors oriented toward the infected cluster, indicating elevated association with the infection condition. These findings are consistent with the known metabolic reprogramming induced by baculovirus infection, which was already discussed. In contrast, pyruvate loading points toward the control group, suggesting preferential pyruvate accumulation or distinct routing in non-infected cells.

##### Heat map

**Fig. 4:**
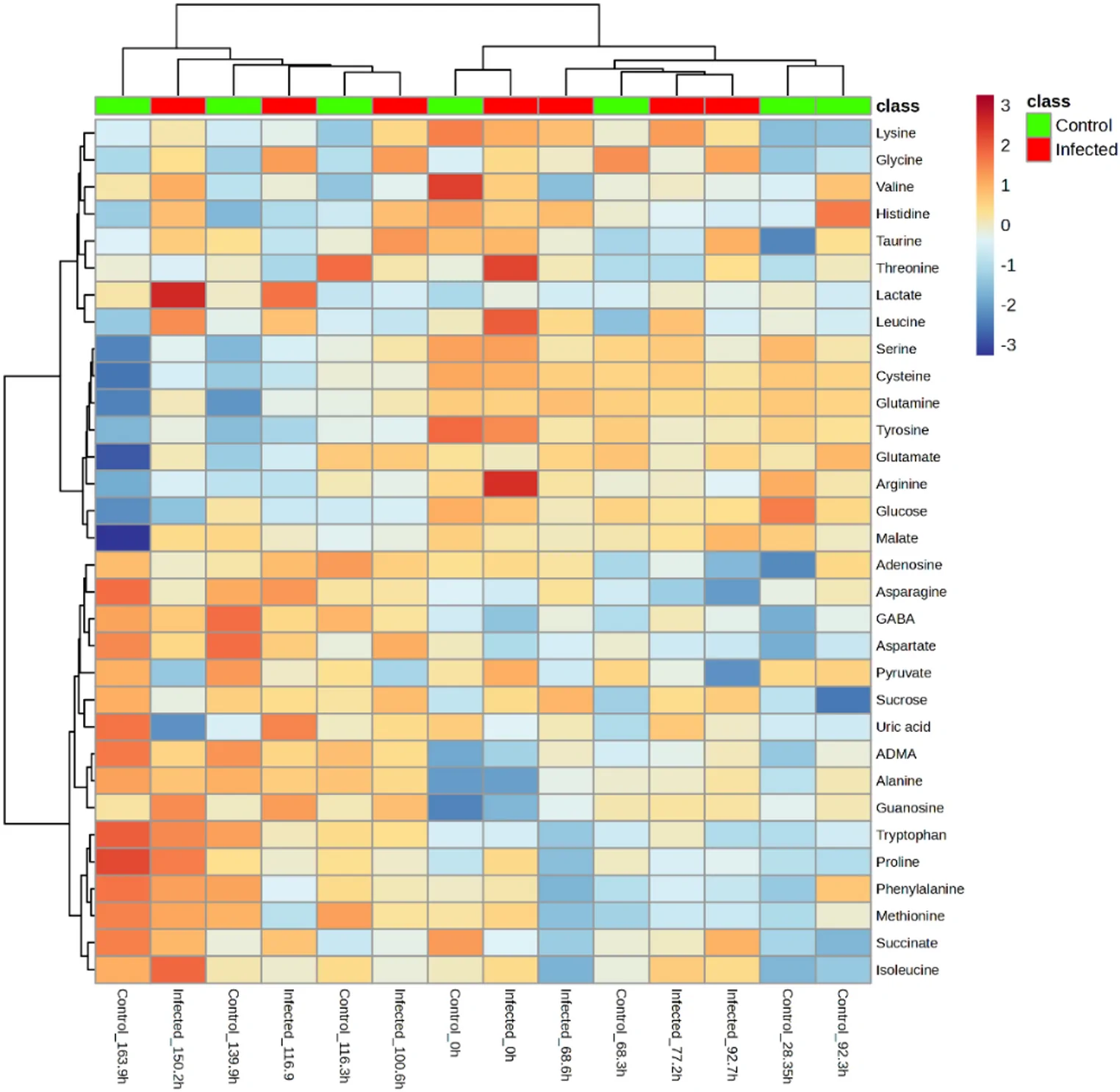
Heatmap of normalized data, measured in Euclidean distance and ward-clustered

The observed behavior in the PCA and PLS-DA biplots was further corroborated by the heatmap (Fig. 4). This map provides an intuitive visualization of the data [65]. Each colored cell on the map corresponds to a concentration value, with samples in columns and features/compounds in rows.

Overall, the samples do not separate perfectly by condition (infected vs. control), but there is partial clustering by time, visualized using the dendrogram. This means that infection is not producing a binary separation in the column clustering. Therefore, metabolic effects exist but are moderate rather than overwhelming. Also, several amino acids show higher relative abundance in controls than in infected samples at later times, consistent with depletion/consumption in infection previously discussed.

##### VIP scores

**Fig. 5:**
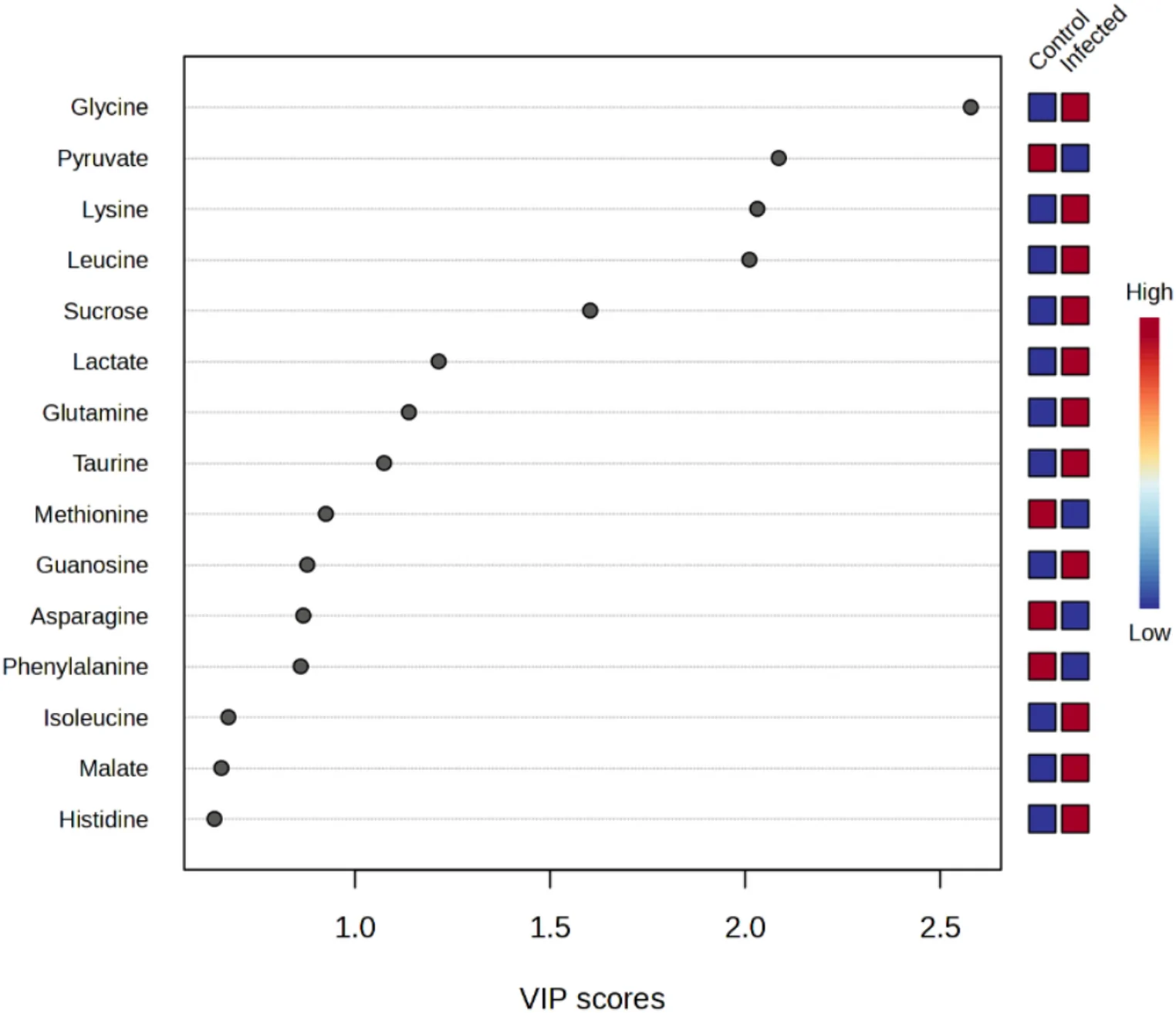
Variable Importance in Projection (VIP) score plot of the metabolites that differed in Infected vs. Control. The colored squares ranging from blue (low) to red (high) indicate the relative abundance of each metabolite

PLS-DA is a supervised technique used to maximize the separation between groups of samples and identify important metabolites [66]. It is ideal for classification and feature selection, using cross-validation to determine the optimal number of components and avoid overfitting [67]. In this paper, the relevance of the PLS-DA is that it is possible to identify metabolites with the greatest potential to enhance the culture medium by calculating their VIP scores. These scores are evaluated from the PLS-DA model, indicating high importance for distinguishing between experimental groups [68] and it is shown in Fig. 5. As previously defined in the Materials and Methods section, only the analytes with a VIP score greater than 1.5 were considered relevant. Therefore, sucrose, leucine, lysine, pyruvate, and glycine were selected to further investigate their potential for supplementation.

Figure 5 shows that the infected group has higher relative abundance of glycine, lysine, leucine, and sucrose than in the non-infected group. Pyruvate was the only relevant analyte that presented the contrary pattern. Again, as in [50] sucrose was marked as a relevant analyte to distinguish between groups, but the reason is the total opposite. Here, the sucrose concentration is much higher in the infected group, indicating a lower consumption rate, not a 3-fold increase. A much smaller increase was already reported in [69], where sucrose consumption indeed slightly rises, but only at the close moment of glucose depletion. This suggests that, in this work’s experiment, glucose depletion may not have been approached, which would explain why no such increase was observed.

Since the pyruvate situation was already addressed in the 3.1 section, a different approach to investigate why glycine, lysine, and leucine appeared as relevant was needed. Following analysis of the recombinant proteins’ primary structures and adjustment to the stoichiometric ratio, the absolute occurrence for each amino acid was determined (Table 5). Among the standard amino acids, leucine was the most frequently required, followed by glycine as the third, and lysine as the eighth, for the combined production of both proteins. The correspondence between the occurrence of these amino acids in the recombinant proteins and their VIP scores may provide a potential association regarding the contribution of recombinant protein synthesis to the extracellular amino-acid profile. However, extracellular amino-acid dynamics are influenced by multiple concurrent processes. Therefore, the observed correspondence should not be interpreted as evidence that the amino-acid composition of the recombinant proteins is the main reason that determines the extracellular environment, nor should the VIP ranking (most important to least) be expected to follow the amino-acid abundance ranking (most used to least) in the recombinant proteins. A quantitative mass-balance analysis incorporating these processes would be required to establish such a relationship.

**Table 5:** Occurrence frequency of each of the 20 standard amino acids individually, considering the 3:2 theoretical expression ratio of G and M rabies structural proteins

Amino acid	Frequency of occurrence (%)
Leu	9.6
Ser	8.0
Gly	7.7
Val	7.3
Thr	6.1
Glu	6.0
Pro	5.9
Lys	5.9
Arg	5.6
Ile	5.4
Asp	5.4
Asn	4.0
Phe	4.0
Tyr	3.2
Ala	3.1
Cys	3.0
Met	2.7
His	2.6
Gln	2.3
Trp	2.1

Rather than providing a direct explanation of the observed metabolic profile, this analysis illustrates how the primary structure of recombinant proteins may be considered as one of several factors when interpreting extracellular amino-acid dynamics during VLP production. This provides a basis for future investigations, particularly studies integrating extracellular metabolomics with quantitative measurements of biomass, protein synthesis, intracellular metabolism, and metabolite fluxes. Such complementary analyses could help to determine the extent to which recombinant protein synthesis contributes to amino-acid demand and could ultimately inform targeted medium supplementation strategies.

Another realistic and valuable next step for this work would be to integrate our data along with findings from comparable studies into a metabolic model focused on central pathways, following the approach in this paper [17]. While building a fully parameterized *Sf9*-specific model would require a substantial collaborative effort, even a partial model could help identify potential bottlenecks in central metabolism during baculovirus infection and VLP production. The proposed model would not necessarily need to be exhaustive to become useful, highlighting which nutrients or feeding strategies, as in previous work [70], are most likely to improve VLP yields, guiding supplementation design with greater mechanistic insight than empirical optimization alone.

Despite providing meaningful new information regarding the extracellular metabolic environment of Sf9 cells during VLP production, this study has some limitations that should be acknowledged. The most important limitation is the absence of biological replicates, with the analysis being based on a total of 14 samples. Consequently, the observed metabolic patterns should be interpreted with caution, and additional experiments with biological replicates would be valuable for assessing the reproducibility of these changes. Furthermore, complementary validation experiments would strengthen this work and provide further support for the biological and engineering implications.

### VLP visualization in transmission electron microscopy and protein quantification

**Fig. 6:**
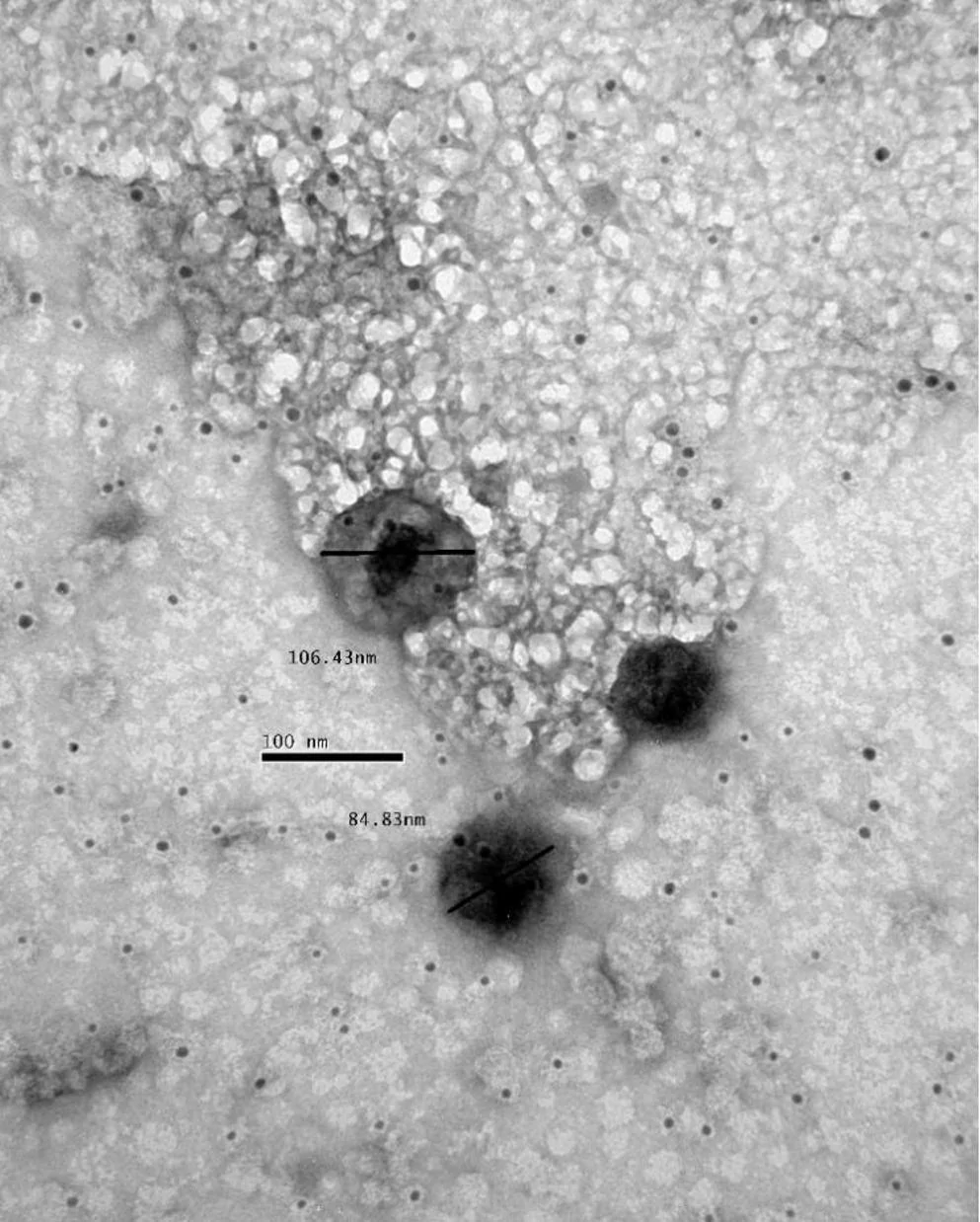
Micrograph with a 200 K magnification confirming production of both inserted recombinant proteins in VLP. The larger black dots are the protein G-specific immunostainings; the smaller ones correspond to the M-specific immunostainings

Transmission electron microscopy (TEM) was employed to conduct a morphological analysis of the produced rabies VLP containing proteins G and M. As shown in Fig. 6, the micrograph confirms the successful formation of rabies VLP in the experiment with infection. The particles exhibited sizes within the range of 107.89 ± 27.41 nm, which aligns with the dimensions previously reported by the research group [38]. Although TEM alone does not provide a complete evaluation of size distribution or population uniformity, the images obtained in this study were adequate to confirm the morphology of the dual-labeled structures and provide an approximate assessment of their characteristic size.

Protein G was quantified by ELISA, reaching a concentration of 92.13 mg/L, whereas Protein M was estimated at 84.46 mg/L through semi-quantitative densitometric analysis performed throughout the infection phase. Both protein concentrations were higher than those previously reported in the literature [38–71], which may be attributed to the optimization of culture conditions, particularly TOI, harvest time, and DO. The differences in the expression levels of these rabies virus proteins may be related to differences in their folding and post-translational processing requirements. In particular, RVGP undergoes glycosylation, involving more complex post-translational processing, whereas RVM does not require the same level of processing [36, 72].

## Conclusions

In conclusion, this study demonstrates that baculovirus infection for Rabies-VLP production induces a clear metabolic reprogramming in Sf9 cells, reflected by dynamic shifts in extracellular nutrient consumption and by-product release throughout the infection process. The combined footprinting and multivariate analyses showed that, although infected and control cultures partially overlapped, infection-specific trends could be identified, with sucrose, leucine, lysine, glycine, and pyruvate emerging as the most relevant discriminating metabolites. These findings, together with the compositional analysis of the recombinant proteins and the TEM confirmation of VLP formation, provide quantitative evidence that the metabolic demand imposed by recombinant expression alters carbon and amino acid utilization in ways that are directly relevant to process performance. Overall, the dataset generated here offers a rational basis for redesigning culture media and developing targeted supplementation or feeding strategies to mitigate metabolic bottlenecks, reduce the cell-density effect, and improve the productivity and robustness of the baculovirus/insect cell platform for VLP manufacture.

## Supplementary Information

Below is the link to the electronic supplementary material.

Supplementary Material 1.

## Data Availability

No datasets were generated or analysed during the current study.
