# Supplementary material for: Footprint metabolomic profile shift of Sf9 insect cell line throughout growth and rabies VLP production
Source: Bioprocess Biosyst Eng. 2026 Sep 22;49(11):3025–41. doi: 10.1007/s00449-026-03428-1 (PMC13661697; doi:10.1007/s00449-026-03428-1)
Supplement: Supplementary file 1 [file 449_2026_3428_MOESM1_ESM.docx]

**Supplementary material**

Contents

[List of Tables	2](#_Toc238111092)

[List of Figures	3](#_Toc238111093)

[Figure 20. Absolute concentrations of Malate (mmol/L) under control and infected conditions…………………………………………………………………………………….14](#_Toc182578323)

[Figure 21. Absolute concentrations of Methionine (mmol/L) under control and infected conditions.	14](#_Toc182578323)

[Figure 22. Absolute concentrations of Phenylalanine (mmol/L) under control and infected conditions.	15](#_Toc182578323)

[Figure 23. Absolute concentrations of Proline (mmol/L) under control and infected conditions…………………………………………………………………………………….15](#_Toc182578323)

[Figure 24. Absolute concentrations of Pyruvate (mmol/L) under control and infected conditions…………………………………………………………………………………….16](#_Toc182578323)

[Figure 25. Absolute concentrations of Serine (mmol/L) under control and infected conditions…………………………………………………………………………………….16](#_Toc182578323)

[Figure 26. Absolute concentrations of Succinate (mmol/L) under control and infected conditions.	17](#_Toc182578323)

[Figure 27. Absolute concentrations of Sucrose (mmol/L) under control and infected conditions…………………………………………………………………………………….17](#_Toc182578323)

[Figure 28. Absolute concentrations of Taurine (mmol/L) under control and infected conditions…………………………………………………………………………………….18](#_Toc182578323)

[Figure 29. Absolute concentrations of Threonine (mmol/L) under control and infected conditions.	18](#_Toc182578323)

[Figure 30. Absolute concentrations of Tryptophan (mmol/L) under control and infected conditions.	19](#_Toc182578323)

[Figure 31. Absolute concentrations of Uric Acid (mmol/L) under control and infected conditions.	19](#_Toc182578323)

[Figure 32. Absolute concentrations of Valine (mmol/L) under control and infected conditions…………………………………………………………………………………….20](#_Toc182578323)

# List of Tables

Table 1. Per–time interval rates of consumption and production in nmol 1$0^{6}$ $cell^{-1}$ $h^{-1}$ for the experiment with infection. Samples were infected at the time 72 h.

|  |  | Time intervals (in hours from the start of the assay) | | | | | | | | | | Time intervals (in hours from the start of the assay) | | | | | | | | | | |
| --- | --- | --- | --- | --- | --- | --- | --- | --- | --- | --- | --- | --- | --- | --- | --- | --- | --- | --- | --- | --- | --- | --- |
| Analyte | 68.66/77.2 | | 77.2/92.75 |  | 92.75/100.58 |  | 100.58/116.95 |  | 116.95/150.6 |  | Analyte | |  | 68.66/77.2 |  | 77.2/92.75 |  | 92.75/100.58 |  | 100.58/116.95 |  | 116.95/150.6 |
| Cys | -0.16 | | -0.54 |  | -0.74 |  | -0.55 |  | 0.15 |  | Guo | |  | 0.08 |  | -0.01 |  | 0.16 |  | 0.11 |  | 0.07 |
| Ast | -6.73 | | -3.38 |  | 9.74 |  | -6.58 |  | 1.24 |  | Tyr | |  | -2.24 |  | -0.50 |  | -2.00 |  | -2.06 |  | 1.19 |
| Asn | -23.09 | | -6.03 |  | 16.06 |  | 1.94 |  | -2.95 |  | A | |  | -0.004 |  | -0.002 |  | 0.009 |  | 0.001 |  | -0.002 |
| Ser | 13.15 | | -14.71 |  | 4.21 |  | -11.52 |  | 2.28 |  | ADMA | |  | -0.02 |  | 0.01 |  | 0.03 |  | 0.002 |  | 0.003 |
| Ala | 34.63 | | 24.13 |  | 44.48 |  | 14.96 |  | 21.88 |  | Ile | |  | 8.98 |  | -2.94 |  | -5.00 |  | -2.84 |  | 4.09 |
| Glu | -24.69 | | 3.38 |  | -1.58 |  | -21.39 |  | 6.94 |  | Leu | |  | -1.60 |  | -7.50 |  | -4.47 |  | 2.09 |  | 2.85 |
| Gly | -2.24 | | 3.24 |  | -0.53 |  | -1.35 |  | -1.24 |  | Phe | |  | 5.77 |  | -2.94 |  | 2.63 |  | -4.64 |  | 4.85 |
| Gln | -147.52 | | -7.36 |  | -76.86 |  | -39.04 |  | 19.41 |  | Trp | |  | 4.81 |  | -3.83 |  | 3.95 |  | -2.69 |  | 2.95 |
| Thr | -9.30 | | 1.91 |  | -2.90 |  | -5.23 |  | 1.81 |  | Tau | |  | -0.23 |  | 0.26 |  | 0.07 |  | -0.40 |  | 0.15 |
| Pro | 9.30 | | -1.47 |  | 0.26 |  | -3.29 |  | 7.71 |  | Pyr | |  | 4.62 |  | -9.05 |  | 5.50 |  | 5.38 |  | -4.12 |
| Lys | 1.60 | | -6.77 |  | -0.53 |  | -5.53 |  | 2.00 |  | Mal | |  | 0.35 |  | 0.56 |  | -2.37 |  | -0.04 |  | 0.34 |
| His | -4.23 | | -0.87 |  | 1.66 |  | -2.71 |  | 1.45 |  | Lac | |  | 1.92 |  | -0.74 |  | -1.32 |  | 6.28 |  | 3.33 |
| Arg | -5.77 | | -3.53 |  | -0.79 |  | -4.94 |  | 1.90 |  | UA | |  | 26.62 |  | -16.63 |  | 12.63 |  | 28.27 |  | -36.11 |
| GABA | 0.01 | | -0.01 |  | 0.02 |  | 0.003 |  | 0.01 |  | Scn | |  | 1.09 |  | 0.43 |  | -1.26 |  | 0.34 |  | 0.11 |
| Val | 3.53 | | -2.94 |  | -2.90 |  | -2.84 |  | 2.85 |  | Suc | |  | -15.07 |  | -1.77 |  | -1.58 |  | -7.78 |  | -0.57 |
| Met | 2.24 | | -2.21 |  | 2.63 |  | -6.58 |  | 5.23 |  | Glc | |  | -36.49 |  | -28.99 |  | -103.73 |  | -48.98 |  | -4.22 |

# List of Figures

Figure 1. Visual result of a three-step normalization applied to the raw data using MetaboAnalyst: Normalized by sum; Log 2 transformation; Range scaling.

Figure 2. Viable cells (Xv) in (cell/mL) under control and infected conditions. Control values are represented by white squares; infected values by black circles.

Figure 3. Cell viability (Cv) in (%) under control and infected conditions. Control values are represented by white squares; infected values by black circles.

Figure 4. Absolute concentrations of Adenosine (mmol/L) under control and infected conditions. Control values are represented by white squares; infected values by black circles.

Figure 5. Absolute concentrations of ADMA (mmol/L) under control and infected conditions. Control values are represented by white squares; infected values by black circles.

Figure 6. Absolute concentrations of Alanine (mmol/L) under control and infected conditions. Control values are represented by white squares; infected values by black circles.

Figure 7. Absolute concentrations of Argigine (mmol/L) under control and infected conditions. Control values are represented by white squares; infected values by black circles.

Figure 8. Absolute concentrations of Asparagine (mmol/L) under control and infected conditions. Control values are represented by white squares; infected values by black circles.

Figure 9. Absolute concentrations of Aspartate (mmol/L) under control and infected conditions. Control values are represented by white squares; infected values by black circles.

Figure 10. Absolute concentrations of Cysteine (mmol/L) under control and infected conditions. Control values are represented by white squares; infected values by black circles.

Figure 11. Absolute concentrations of GABA (mmol/L) under control and infected conditions. Control values are represented by white squares; infected values by black circles.

Figure 12. Absolute concentrations of Glucose (mmol/L) under control and infected conditions. Control values are represented by white squares; infected values by black circles.

Figure 13. Absolute concentrations of Glutamate (mmol/L) under control and infected conditions. Control values are represented by white squares; infected values by black circles.

Figure 14. Absolute concentrations of Glutamine (mmol/L) under control and infected conditions. Control values are represented by white squares; infected values by black circles.

Figure 15. Absolute concentrations of Glycine (mmol/L) under control and infected conditions. Control values are represented by white squares; infected values by black circles.

Figure 16. Absolute concentrations of Guanosine (mmol/L) under control and infected conditions. Control values are represented by white squares; infected values by black circles.

Figure 17. Absolute concentrations of Histidine (mmol/L) under control and infected conditions. Control values are represented by white squares; infected values by black circles.

Figure 18. Absolute concentrations of Isoleucine (mmol/L) under control and infected conditions. Control values are represented by white squares; infected values by black circles.

Figure 19. Absolute concentrations of Lactate (mmol/L) under control and infected conditions. Control values are represented by white squares; infected values by black circles.

Figure 20. Absolute concentrations of Leucine (mmol/L) under control and infected conditions. Control values are represented by white squares; infected values by black circles.

Figure 21. Absolute concentrations of Lysine (mmol/L) under control and infected conditions. Control values are represented by white squares; infected values by black circles.

Figure 22. Absolute concentrations of Malate (mmol/L) under control and infected conditions. Control values are represented by white squares; infected values by black circles.

Figure 23. Absolute concentrations of Methionine (mmol/L) under control and infected conditions. Control values are represented by white squares; infected values by black circles.

Figure 24. Absolute concentrations of Phenylalanine (mmol/L) under control and infected conditions. Control values are represented by white squares; infected values by black circles.

Figure 25. Absolute concentrations of Proline (mmol/L) under control and infected conditions. Control values are represented by white squares; infected values by black circles.

Figure 26. Absolute concentrations of Pyruvate (mmol/L) under control and infected conditions. Control values are represented by white squares; infected values by black circles.

Figure 27. Absolute concentrations of Serine (mmol/L) under control and infected conditions. Control values are represented by white squares; infected values by black circles.

Figure 28. Absolute concentrations of Succinate (mmol/L) under control and infected conditions. Control values are represented by white squares; infected values by black circles.

Figure 29. Absolute concentrations of Sucrose (mmol/L) under control and infected conditions. Control values are represented by white squares; infected values by black circles.

Figure 30. Absolute concentrations of Taurine (mmol/L) under control and infected conditions. Control values are represented by white squares; infected values by black circles.

Figure 31. Absolute concentrations of Threonine (mmol/L) under control and infected conditions. Control values are represented by white squares; infected values by black circles.

Figure 32. Absolute concentrations of Tryptophan (mmol/L) under control and infected conditions. Control values are represented by white squares; infected values by black circles.

Figure 33. Absolute concentrations of Uric Acid (mmol/L) under control and infected conditions. Control values are represented by white squares; infected values by black circles.

Figure 34. Absolute concentrations of Valine (mmol/L) under control and infected conditions. Control values are represented by white squares; infected values by black circles.
